# The Association of P2Y_12_ Inhibitor Pretreatment With Length of Stay Among Patients With Acute Coronary Syndrome Who Underwent Coronary Artery Bypass Graft Surgery: A Cohort Study

**DOI:** 10.1155/cdr/8959128

**Published:** 2025-04-08

**Authors:** Kramer J. Wahlberg, Cyrus Thomas-Walker, Bradley J. Tompkins, Juvena Hitt, Allen B. Repp, William Hopkins

**Affiliations:** ^1^Department of Medicine, The Larner College of Medicine at the University of Vermont, Burlington, Vermont, USA; ^2^Department of Medicine, The University of Vermont Medical Center, Burlington, Vermont, USA

**Keywords:** CABG, cost, length of stay, P2Y_12_ inhibitor, pretreatment

## Abstract

**Introduction:** Recent clinical practice guidelines do not recommend routine P2Y_12_ inhibitor pretreatment for patients with non-ST-elevation acute coronary syndromes (NSTE-ACS) treated with an early invasive strategy based upon clinical trial data suggesting no improvement in clinical outcomes and increased risk of bleeding. A subset of patients with NSTE-ACS who receive pretreatment and subsequently require coronary artery bypass graft (CABG) surgery may require lengthy P2Y_12_ inhibitor washout to reduce periprocedural bleeding risk, potentially prolonging hospitalization and increasing costs. We sought to study the association of P2Y_12_ inhibitor pretreatment on value-based outcomes including length of stay, cost, and discharge destination.

**Methods:** We conducted a retrospective cohort study of patients presenting with NSTE-ACS who underwent CABG at a tertiary academic medical center between 2019 and 2021. We assessed the frequency of P2Y_12_ inhibitor pretreatment over the study period and compared risk-adjusted length of stay, cost of hospitalization, and discharge destination among patients who did or did not receive pretreatment.

**Results:** One hundred eighty-eight patients met inclusion criteria, and 77% received pretreatment. The rate of pretreatment decreased significantly over the study period (*p* < 0.001). Pretreatment was associated with longer preoperative length of stay (4.2 ± 1.6 vs. 3.4 ± 2.5 days, *p* = 0.019), with no significant difference in postoperative or total length of stay. There was no difference in cost of hospitalization or likelihood of discharge to home following CABG.

**Conclusion:** Among patients presenting with NSTE-ACS who underwent inpatient CABG, P2Y_12_ inhibitor pretreatment was associated with longer preoperative length of stay, but no difference in total length of stay, cost of hospitalization, or discharge destination in this observational, single-center study.

## 1. Introduction

Dual antiplatelet therapy (DAPT) consisting of aspirin and a P2Y_12_ inhibitor reduces the incidence of cardiovascular death and myocardial infarction in patients with non-ST-elevation acute coronary syndrome (NSTE-ACS) [1]. Among patients with NSTE-ACS managed with an early invasive approach, the P2Y_12_ inhibitor was historically administered at the time of diagnosis and upstream from cardiac catheterization, often referred to as pretreatment, with the theoretical goal of minimizing periprocedural ischemic complications through achieving adequate platelet inhibition prior to percutaneous coronary intervention (PCI). However, contemporary studies of P2Y_12_ inhibitor pretreatment have shown no significant reduction in periprocedural thrombotic complications among patients treated with an invasive approach, with some suggestion of higher rates of bleeding [2–10].

Adverse events related to DAPT are particularly relevant to the upwards of 35% of patients initially diagnosed with NSTE-ACS who undergo coronary angiography and do not receive PCI, including the 6–13% who are referred for urgent, in-hospital coronary artery bypass graft (CABG) [2, 5, 7, 11]. Pretreatment may result in delayed CABG to allow time for P2Y_12_ inhibitor washout [12, 13], which may take up to 5–7 days. Furthermore, perioperative DAPT is associated with increased risks of major bleeding, transfusion, reoperation, and longer hospital stay [14–22].

Increasingly, guideline committees highlight the incorporation of cost and value of care into recommendations [23], and value-based healthcare payment systems incentivize high-quality care with reduced costs. In a provocative review in the *Journal of Hospital Medicine*, authors identified routine P2Y_12_ inhibitor pretreatment in NSTE-ACS as one of the “Things We Do for No Reason,” [24] suggesting it is a low-value care practice. With the potential for P2Y_12_ inhibitor pretreatment to lead to increased periprocedural bleeding and delayed surgical revascularization when indicated, we sought to understand the relationship between P2Y_12_ inhibitor pretreatment and value-based outcomes such as length of stay, cost of care, and discharge destination among patients who subsequently underwent CABG surgery. We hypothesized that P2Y_12_ inhibitor pretreatment would lead to longer length of stay, lower likelihood of discharge directly to home, and increased cost of care in this group of patients.

## 2. Methods

We performed a retrospective cohort study of all patients undergoing CABG at our institution between 2019 and 2021. The cardiothoracic surgery program at our rural, tertiary academic medical center performed on average 257 CABG surgeries per year during the study period. The manuscript was prepared in accordance with the Strengthening the Reporting of Observational Studies in Epidemiology (STROBE) guidelines [25]. Completed STROBE checklist is included as supplementary material. Among patients undergoing CABG who presented with NSTE-ACS, our objective was to determine the frequency of P2Y_12_ inhibitor pretreatment over time and the association of pretreatment with length of stay, cost of hospitalization, and discharge destination. A secondary outcome was major bleeding as assessed by the Bleeding Academic Research Consortium (BARC) classification system [26]. Patients were identified by CABG procedure codes and included if discharged between January 1, 2019, and December 31, 2021. Patients were excluded if the following criteria were met after chart review: reason for admission other than NSTE-ACS, cardiac catheterization not performed at our institution and/or during same hospitalization as CABG, DAPT treatment prior to admission, prior CABG, or concomitant valve surgery at the time of CABG. Patient demographics, length of stay, and discharge destination were obtained directly from the medical record. Length of stay was measured by number of midnights and divided into preoperative, postoperative, and total length of stay. Preoperative length of stay was defined as time from cardiac catheterization to CABG, as the inherent variability in time from admission to cardiac catheterization is confounded by factors including initial admission to partner hospital awaiting transfer to PCI center and catheterization laboratory availability. Total length of stay was defined as time from cardiac catheterization to discharge. Total cost of hospitalization was obtained from the institution's cost-accounting system and represents the sum of hospital's direct and indirect costs for each hospital encounter.

As timing of P2Y_12_ inhibitor administration in relation to catheterization was fundamental to the research question, a manual chart review was required to determine whether a patient received pretreatment and to collect other variables including reason for admission, Society of Thoracic Surgeons (STS) risk scores, and bleeding events. STS scores [27] are generated from a guideline-recommended and validated risk calculator used to predict procedure-specific short- and long-term risks in multiple domains among patients undergoing cardiac surgery. The risk calculator integrates numerous patient and procedure characteristics including demographics, comorbid conditions, medications, laboratory values, coronary angiography findings, cardiopulmonary status, and planned surgery. At our institution, STS scores were routinely calculated in real time and documented during the preoperative cardiothoracic surgery consultation, allowing for unbiased extraction by retrospective chart review. Manual chart review was systematically performed by two investigators using a chart abstraction tool developed in REDCap [28, 29]. The two investigators independently reviewed a random sample of overlapping charts, and interrater agreement was determined to be 97.7% (kappa statistic 0.96).

### 2.1. Statistical Analysis

Prior to the study, a power calculation was performed. Based upon an institutional estimate of mean 10-day length of stay for patients undergoing left heart catheterization and CABG, it was determined that 240 patients would be needed to detect a 20% (2-day) difference in length of stay. The frequency of P2Y_12_ inhibitor pretreatment was expressed as a percentage of patients receiving pretreatment per month and analyzed using a linear regression model. Analysis of variance was used to assess for differences in length of stay for pretreated patients, multivariate linear regression for differences in cost of hospitalization, and multivariable logistic regression for discharge destination (home vs. rehabilitation facility) and bleeding outcomes. Risk adjustments were made based upon STS score. Postoperative and total length of stay were adjusted using the STS long length of stay risk. Cost of hospitalization, discharge destination, and bleeding outcomes were adjusted using the STS morbidity and mortality risk. No adjustments were made for preoperative length of stay, as the STS score is validated for postoperative risk assessment. The power analysis and all statistical analyses were performed using Stata software (Stata 16.1, StataCorp, LLC, College Station, Texas). A *p*-value cutoff of < 0.05 was considered to determine statistical significance in this analysis.

### 2.2. Exploratory Analysis

During the study period in February 2021, an institutional policy was enacted recommending against routine pretreatment of *outpatients* presenting for elective coronary angiography. With the assumption that this attention to pretreatment for outpatients could affect decisions to pretreat inpatients with NSTE-ACS, we performed a prespecified exploratory analysis among inpatients and stratified pretreatment before and after enactment of the outpatient pretreatment policy.

### 2.3. Ethical Considerations

The Research Protections Office at the University of Vermont determined that the project was exempt from review under Exemption Category 4 (secondary research on data or specimens).

## 3. Results

**Figure 1:**
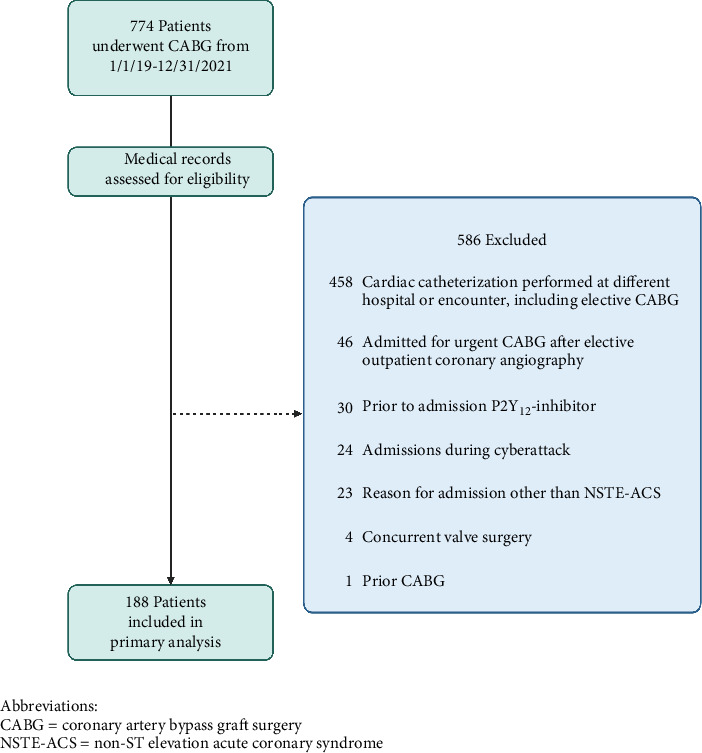
Flow diagram. Abbreviations: CABG = coronary artery bypass graft surgery, NSTE-ACS = non-ST-elevation acute coronary syndrome.

Seven hundred seventy-four patients underwent CABG between 2019 and 2021, and 188 were included in the study. The predominant reason for exclusion was preoperative cardiac catheterization not performed at our institution or during the same hospitalization as CABG and initial presentation other than NSTE-ACS such as an elective outpatient cardiac catheterization (Figure 1). Age, gender, race, payer, and STS scores were similar between patients who did and did not receive pretreatment (Table 1).

### 3.1. Frequency of P2Y_12_ Inhibitor Pretreatment

**Figure 2:**
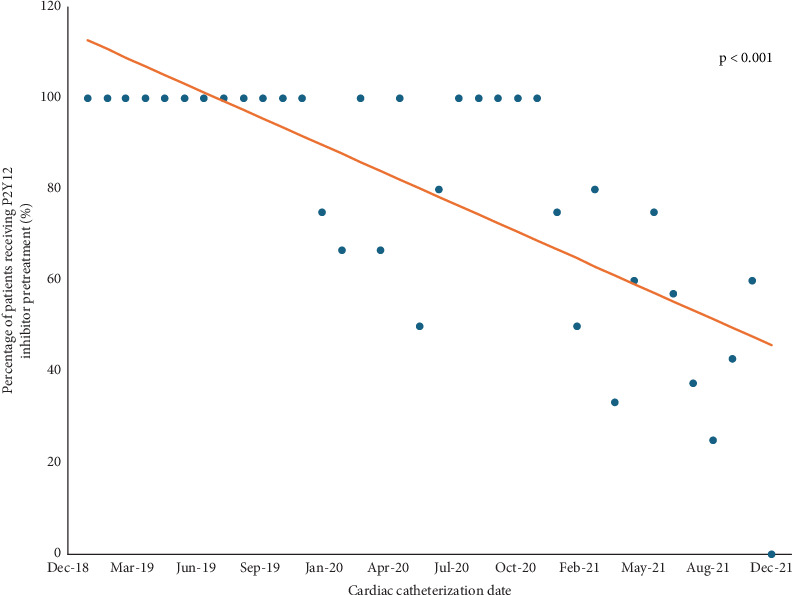
Percentage of patients receiving P2Y_12_ inhibitor pretreatment per month of the study period. Line represents trend over time via linear regression.

Of the 188 patients included in the primary analysis, 145 (77%) received P2Y_12_ inhibitor pretreatment. Clopidogrel was the most common P2Y_12_ inhibitor (52.4%) used for pretreatment, followed by ticagrelor (46.9%). The frequency of pretreatment decreased throughout the study period, with the date of cardiac catheterization significantly associated with the use of pretreatment (*R*^2^ = 0.55, *p* < 0.001) as shown in Figure 2.

### 3.2. Association of P2Y_12_ Inhibitor Pretreatment With Length of Stay, Cost of Hospitalization, and Discharge Destination

Patients who received P2Y_12_ inhibitor pretreatment were observed to have a longer mean preoperative length of stay than those who did not receive pretreatment (4.2 ± 1.6 vs. 3.4 ± 2.5 days, *p* = 0.019; Table 2). However, there was no significant difference in adjusted postoperative (*p* = 0.15) or total length of stay (*p* = 0.91) when adjusted for STS long length of stay risk score. After adjustment for STS morbidity and mortality risk, pretreatment was not associated with a difference in total cost of hospitalization (*p* = 0.173) or with the likelihood of being discharged to home (OR 0.85, 95% CI 0.3–2.3, *p* = 0.746).

### 3.3. Bleeding Complications

There was a low rate of major bleeding as defined by BARC 3–5 bleeding events and no significant association of P2Y_12_ inhibitor pretreatment with major bleeding (OR 1.02, 95% CI 0.2–5.2).

### 3.4. Exploratory Analysis

A prespecified exploratory analysis of the frequency of pretreatment for inpatients with acute coronary syndrome before and after the enactment of an institutional policy pertaining to pretreatment of outpatients found a significant change in the percentage of inpatients who received pretreatment during these two time periods (93% received pretreatment before February 2021 vs. 49% after, *p* < 0.001, Table 3).

## 4. Discussion

DAPT is a fundamental component of the medical treatment of NSTE-ACS; however, emerging data and guidelines [30] have questioned the practice of routine upstream DAPT pretreatment for patients with NSTE-ACS who undergo coronary angiography as part of an invasive management approach in whom the coronary anatomy is unknown. Pretreatment has implications for patients subsequently found to have multivessel coronary artery disease and referred for urgent CABG, as they will often undergo a lengthy washout period to avoid the risk of increased perioperative bleeding [11, 12]. This retrospective institutional study of patients who underwent CABG demonstrates that P2Y_12_ inhibitor pretreatment is occurring less frequently over time at our institution and is associated with a longer preoperative length of stay among patients that undergo CABG.

Despite accumulating evidence questioning the benefit of routine pretreatment in NSTE-ACS [31] and the 2020 European Society of Cardiology guidelines recommending against routine pretreatment (Class 3 recommendation) for most patients with NSTE-ACS managed with an early invasive strategy [30], the practice remains common and varies significantly by institution and geography [32]. Meta-analyses suggest that practice is changing over time, and Dworeck et al. analyzed registry data from Sweden demonstrating that local policy can be very effective in changing clinical practice with a decrease from 99% to 15% pretreatment following a recommendation against routine pretreatment [3]. On the contrary, there was no policy guiding routine P2Y_12_ inhibitor pretreatment for NSTE-ACS at our institution, with the decision to pretreat left up to the treating cardiologist's discretion during the study period. Nonetheless, we observed a significant change in practice during the study period wherein > 90% of patients presenting with NSTE-ACS who underwent CABG received pretreatment in the early study period while < 50% of patients received pretreatment by the end of the study period. There are several potential explanations for this change in practice. First, it is likely reflective of the emerging evidence and guideline recommendations against routine pretreatment. Similarly, in February 2021, an institutional policy was enacted to stop routine pretreatment of outpatients presenting for elective coronary angiography. Although this was a different patient population than patients included in this study, who presented with NSTE-ACS, the outpatient policy stimulated conversation regarding pretreatment, which may have led to heightened awareness towards this topic and influenced decision-making for all patients.

P2Y_12_ inhibitor pretreatment is often cited as a reason for delaying surgery, although this has not been specifically studied in a controlled fashion. Contemporary randomized clinical trials investigating P2Y_12_ inhibitor pretreatment have predominantly included patients with NSTE-ACS managed with an early invasive approach who, on average, underwent coronary angiography within 24 h [5, 10]. In these trials, the primary composite endpoints consisted of clinical outcomes including cardiovascular death, myocardial infarction, stroke, periprocedural ischemic complications such as stent thrombosis, and bleeding. Length of stay and cost were not typically reported. Furthermore, 6–7% of patients in these trials underwent CABG, and it is unclear what effect, if any, pretreatment had on value-based outcomes for these patients. To that end, this study provides single-center, observational-level data describing the association of P2Y_12_ inhibitor pretreatment with cost and length of stay.

**Figure 3:**
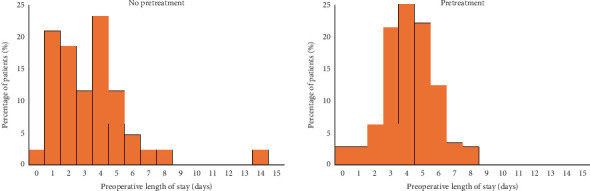
Histogram depicting length of stay from cardiac catheterization to coronary artery bypass graft surgery among patients who did and did not receive P2Y_12_ inhibitor pretreatment.

Observational studies have demonstrated an association with preoperative exposure to P2Y_12_ inhibitors and increased risk of perioperative bleeding and length of stay [17, 19–22]. Given the increased bleeding risk, patients who receive preoperative DAPT often wait for the P2Y_12_ inhibitor to washout prior to surgery. The 2014 American College of Cardiology and American Heart Association guidelines for management of NSTE-ACS recommend P2Y_12_ inhibitor treatment (Class 1, level of Evidence B) for patients undergoing an invasive or ischemia-guided strategy but do not specify timing of administration [1], whereas the 2020 European Society of Cardiology guidelines for management of ACS recommend against routine P2Y_12_ inhibitor pretreatment (Class III, level of Evidence A), perhaps reflecting more recent trial data [30]. Registry data from 2015 [11] showed that the majority of patients presenting with NSTE-ACS who underwent CABG went to the operating room after 48 h. It should be noted that these data were from a time when pretreatment was more common and prior to contemporary clinical trials or guidelines that recommended against routine pretreatment. Nonetheless, our data show similar trends. While more patients who did not receive pretreatment went for CABG on hospital Days 1 or 2 than those who were pretreated (Figure 3), most patients went to the operating room after 48 h. Lastly, platelet function testing has the potential to guide decision-making regarding the timing of cardiac surgery in patients who receive P2Y_12_ inhibitors. This preoperative testing was not routinely performed during the study period and therefore not included in the present study.

Typically, most stable patients with NSTE-ACS who receive P2Y_12_ inhibitor pretreatment without an indication for emergent CABG will undergo a lengthy preoperative washout resulting in delayed surgery, and those who go more urgently to the operating room following pretreatment may be exposed to increased bleeding risk. Therefore, it is reasonable to expect that patients who received P2Y_12_ inhibitor pretreatment would experience longer hospital stays primarily driven by longer preoperative length of stay. Longer hospitalization may plausibly lead to increased cost and possibly patient deconditioning, resulting in a lower likelihood of discharge directly to home following CABG. In this study, we observed an approximately one-midnight longer length of stay from the time of cardiac catheterization to surgery, with no change in postoperative length of stay. The small difference in preoperative length of stay did not translate into a difference in overall length of stay, although our study was underpowered to detect the hypothesized 2-day difference in length of stay. The change in preoperative length of stay could be attributed to pretreatment, as histograms in Figure 3 show that patients who did not receive pretreatment were more likely to go for CABG within the first 72 h after coronary angiography. However, there were still many patients who did not receive pretreatment that waited 5 days or longer for surgery, suggesting that other factors unrelated to pretreatment impacted length of stay such as operating room, surgeon, or staff availability.

### 4.1. Limitations

This study is limited by its single-center, observational design. While attempts were made to adjust for individual patient factors using the comprehensive STS score calculated at the time of surgery, there are unmeasured variables that confound the findings, and our results are hypothesis-generating. The study was conducted at a rural, tertiary academic medical center, and the study population lacked gender and racial diversity, potentially limiting generalizability of the results. Notably, female patients and non-White patients were underrepresented. Additionally, uncontrolled factors at our institution that may have influenced length of stay during the study period include challenges with census and staffing during the COVID-19 pandemic, a cyberattack with transient outage of the electronic medical record, and unexpected operating room shortages. The small sample size of this cohort study rendered the analysis sensitive to outlier effects. Specifically, one preoperative length of stay among the patients who did not receive pretreatment (Figure 3) skewed the mean length of stay for this group with a preoperative length of stay of nearly 15 days as compared to the mean of 3.4 days for the cohort. Lastly, Figure 1 describes that a significant proportion of screened patients were excluded from the analysis. This is largely due to beginning with a screened population that included all patients undergoing CABG, which includes many patients who do not present with NSTE-ACS and therefore not relevant to this study question. Furthermore, as we were interested in associations with P2Y_12_ inhibitor pretreatment, it was necessary to exclude patients who did not undergo cardiac catheterization and CABG during the same admission.

This study investigated only patients who ultimately underwent CABG and did not include all patients presenting with NSTE-ACS who may have otherwise been treated with PCI or medical therapy. It is possible that the inherent differences between patients who did and did not ultimately undergo CABG populations impacted the treating clinician's decision to pretreat, although the tendency is to avoid P2Y_12_ inhibitor pretreatment in those with a high index of suspicion for multivessel disease requiring surgical revascularization. Furthermore, in an attempt to isolate the impact of pretreatment, we defined preoperative length of stay as the time from cardiac catheterization to surgery. This does not consider the length of stay from time of admission to referring hospital or our institution and cardiac catheterization, which may influence the decision to pretreat for some patients. For example, patients with an anticipated delay to cardiac catheterization due to factors such as waiting for transfer to a PCI center are not reflective of the pretreatment clinical trial population and may stand to benefit from pretreatment. Importantly, while preoperative length of stay for this study did not include the precatheterization length of stay, the total cost of hospitalization did include any potential precatheterization hospital days at our institution.

Lastly, while our motivation was to investigate the impact of pretreatment on value-based outcomes, this small, single-center study is not adequate to make a value proposition, and further study is needed with larger, more diverse patient populations.

## 5. Conclusion

Among patients presenting with NSTE-ACS who ultimately underwent inpatient CABG, P2Y_12_ inhibitor pretreatment was associated with a longer preoperative length of stay, but no difference in total length of stay, cost of hospitalization, or discharge destination in this observational, single-center study. Larger, multicenter studies are needed to analyze routine P2Y_12_ inhibitor pretreatment and its impact on resource utilization.

## Figures and Tables

**Table 1:** Characteristics of patients who did and did not receive P2Y_12_ inhibitor pretreatment.

	**Patients receiving pretreatment (** **n** = 145**)**	**Patients not receiving pretreatment (** **n** = 43**)**	**p** **-value**
Age, median (IQR)	66.7 (58.9–73.3)	67.3 (54.7–75.3)	0.91
Female gender, # (%)	27 (18.6)	9 (20.9)	0.74
Race/ethnicity^^^ (%)			
White, non-Hispanic	131 (95.6)	38 (95.0)	0.06
Black, non-Hispanic	1 (0.7)	0 (0.0)	
Asian/other/unknown, non-Hispanic	5 (3.7)	0 (0.0)	
Hispanic	0 (0.0)	2 (5.0)	
Payer, # (%)			
Commercial	41 (28.3)	10 (23.3)	0.59
Medicare	80 (55.2)	23 (53.5)	
Medicaid	15 (10.3)	7 (16.3)	
Self-pay	1 (0.7)	1 (2.3)	
Other	8 (5.5)	2 (4.7)	
STS risk, mean (SD)			
STS mortality risk	1.6 (2.0)	1.9 (2.2)	0.39
STS morbidity & mortality risk	10.8 (10.7)	11.5 (8.2)	0.32
STS long length of stay risk⁣^∗^	4.2 (5.6)	4.6 (4.8)	0.43
Discharge disposition: home, # (%)	122 (81.4)	35 (84.1)	0.67

*Note:* Continuous variables were evaluated using Wilcoxon rank sum tests and categorical variables via Chi-square or Fisher's exact test.

**Table 2:** Comparison of length of stay, cost of hospitalization, and discharge disposition among patients who did and did not receive P2Y_12_ inhibitor pretreatment.

**Length of stay (days), mean (SE)**	**Patients receiving pretreatment (** **n** = 145**)**	**Patients not receiving pretreatment (** **n** = 43**)**	**p** **-value**

Preoperative length of stay	4.2 (0.2)	3.4 (0.3)	0.019
Postoperative length of stay⁣^∗^	6.2^^^ (0.5)	7.0 (0.3)	0.15
Total length of stay⁣^∗^	10.3^^^ (0.6)	10.4 (0.3)	0.91

⁣^∗^Adjusted for Society of Thoracic Surgeons (STS) long length of stay risk.

^^^ *n* = 144 for postoperative and total length of stay pretreatment cohort as one patient did not have a STS risk score available for adjustment.

**Table 3:** Exploratory analysis of P2Y_12_ inhibitor pretreatment before and after February 1, 2021.

**Period**	**Patients receiving pre-treatment**	**Patients not receiving pre-treatment**	**p** ** -value**
	***n* (%)**	***n* (%)**	
Before Feb 1, 2021 (*n* = 121)	112 (93)	9 (7)	< 0.001
After Feb 1, 2021 (*n* = 67)	33 (49)	34 (51)	

*Note:* Chi-square analysis of frequency of P2Y_12_ inhibitor pretreatment before and after February 1, 2021, shows that practice varies significantly during these time periods. February 1, 2021 was chosen as this date corresponds with the enactment of an institutional policy stopping routine P2Y_12_ inhibitor pretreatment for patients undergoing outpatient coronary angiography. However, it was hypothesized that this change in practice may have influenced the decision to pretreat the inpatients included in this study.

## Data Availability

Study data will be made available by the authors upon a reasonable request.
